# Genome-Wide Identification of MicroRNAs and Immune-Related Proteins Provides Insights into Antiviral Adaptations in Common Vampire Bat

**DOI:** 10.3390/ani15213063

**Published:** 2025-10-22

**Authors:** Yicheng Yan, Tianyi Liu, Xiaopeng He, Mingdao Mu, Zhiyuan Yang

**Affiliations:** 1 School of Artificial Intelligence, Hangzhou Dianzi University, Hangzhou 310018, China; 2 School of Medicine, Southeast University, Nanjing 210009, China

**Keywords:** *Desmodus rotundus*, non-coding RNA, interferon regulatory factor, comparative genomic analysis, antiviral mechanisms

## Abstract

**Simple Summary:**

The common vampire bat (*Desmodus rotundus*) harbors many viruses but rarely develops disease, suggesting unique immune strategies. In this study, we combined genomic comparison, protein annotation, and microRNA analysis to investigate its antiviral mechanisms. We identified conserved interferon regulators, immune-related proteins, and novel miRNAs targeting genes involved in apoptosis, stress response, and infection pathways. Network analysis further highlighted key hub genes controlling immune balance. Our findings show that *D. rotundus* relies on finely tuned gene regulation to tolerate viruses without pathology.

**Abstract:**

Bats are natural reservoirs for diverse viruses, yet they rarely develop disease, suggesting unique antiviral adaptations. In this study, we performed a comprehensive genome-wide analysis in the common vampire bat (*Desmodus rotundus*), integrating comparative genomics, functional annotation, microRNA (miRNA) discovery, target prediction, and network-based analyses. Comparative genomic analysis revealed that *Phyllostomus discolor* exhibits the highest protein homology (97.4%) with *D. rotundus*. Alignment of interferon regulatory factors (IRFs) indicated strong conservation of IRF1, IRF5, and IRF8, while IRF4 and IRF7 showed divergence, reflecting bat-specific modulation of interferon signaling. Functional annotation of previously uncharacterized proteins identified immune-related elements, including toll-like receptor 4, syncytin-1, and endogenous retroviral sequences, highlighting the integration of viral components into host immunity. We further identified 19 novel miRNAs in *D. rotundus*, with high-confidence target genes such as *SOD2*, *TRIM28*, and *FGFR1* involved in antiviral defense, apoptosis regulation, and oxidative stress response. Functional enrichment analyses revealed processes associated with wound healing, apoptosis suppression, infection response, and longevity. Network entropy analysis highlighted central regulatory hubs, including *MYC*, *BCL2*, and *KIF1B*, influencing cell cycle, survival, and immune balance. Collectively, these results demonstrate that *D. rotundus* employs an integrated regulatory network combining conserved immune factors, lineage-specific gene divergence, and miRNA-mediated fine-tuning to achieve viral tolerance without pathology. This study expands our understanding of bat antiviral biology and provides candidate molecular targets for future functional and translational research.

## 1. Introduction

Bats, belonging to the order Chiroptera, exhibit several unique physiological and immunological characteristics compared to other mammals. *Desmodus rotundus*, commonly known as the common vampire bat, is a leaf-nosed species that feeds exclusively on the blood of mammals [1]. This species possesses notable adaptations such as powered flight and an unusually long lifespan for its body size [2]. Importantly, *D. rotundus* serves as an asymptomatic reservoir for numerous pathogenic viruses that are often lethal to other mammals. Multiple viruses, including coronaviruses, have been previously identified in *D. rotundus*, highlighting the species-specific nature of bat–virus interactions [3]. Therefore, understanding the antiviral mechanisms in *D. rotundus* is of great clinical relevance for improving the prevention, diagnosis, and control of zoonotic viral infections.

Research on bat antiviral immunity has revealed that many bat species, including *D. rotundus*, possess enhanced innate immune defenses that allow them to tolerate high viral loads without developing overt disease. For example, certain bat lineages display constitutive expression of type I and III interferons, which prime downstream interferon-stimulated genes (ISGs) even in the absence of infection [4]. In addition, bats often exhibit attenuated inflammatory signaling, such as dampened NLRP3 inflammasome activation, reducing immunopathology during viral replication [5]. Although specific functional studies on *D. rotundus* remain limited, transcriptomic analyses suggest that vampire bats may similarly leverage a tightly regulated interferon response—balancing viral clearance with minimal tissue damage. Moreover, the RNA interference (RNAi) pathway in bats appears to be more active than in many other mammals, potentially contributing to post-transcriptional suppression of viral genomes [6]. Collectively, these mechanisms enable *D. rotundus* to act as an asymptomatic viral reservoir, maintaining persistent infections while avoiding the pathological consequences observed in other hosts.

The latest assembled genome version (HLdesRot8A.1) of *D. rotundus* was released by Blumer et al. [7] and the latest annotation release GCF_022682495.2 was published in February 2025. However, a considerable number of proteins in this genome remain annotated only as “uncharacterized proteins” (UCPs). Traditional annotation methods rely on similarity searches against the non-redundant (nr) reference database, which itself contains many UCPs, complicating accurate functional annotation. Because previous studies indicate UCPs can contribute to species-specific adaptations and evolution [8], we hypothesize that some uncharacterized proteins in *D. rotundus* contribute to antiviral responses. Identifying and characterizing these proteins may thus uncover novel antiviral factors and expand our understanding of the bat proteome.

In addition, the role of microRNAs (miRNAs) in *D. rotundus* remains largely unexplored. MiRNAs are small non-coding RNAs (ncRNAs) and are typically 18–25 nucleotides in length. These molecules play critical roles in regulating immune responses and have been widely implicated in antiviral defense, including the suppression of viral replication [9]. Recent work by Dai et al. [6] demonstrated that small interfering RNAs (siRNAs) produced in bats can guide the RNA-induced silencing complex (RISC) to specifically degrade viral genomes, offering a low-inflammatory and energy-efficient antiviral strategy. Given the structural and functional similarities between siRNAs and miRNAs, we hypothesize that miRNAs may also play a key role in the antiviral mechanisms of *D. rotundus*. Advances in bioinformatics have facilitated the discovery of novel miRNAs from transcriptomic data in various non-model organisms, such as *Mentha piperita* [10] and *Wuchereria bancrofti* [11], suggesting that similar approaches can be effectively applied to study miRNA-mediated antiviral regulation in *D. rotundus*.

In this study, we performed a genome-wide analysis to identify novel proteins and miRNAs in *D. rotundus*. We conducted functional annotation of previously uncharacterized proteins and investigated their potential antiviral roles. In parallel, species-specific miRNAs were identified, and their possible functions in immune regulation were explored. Our findings provide new insights into the molecular basis of viral tolerance in bats and may contribute to the broader understanding of host–virus coevolution.

## 2. Materials and Methods

### 2.1. Protein Comparison with Other Bats

The latest genome annotation of *D. rotundus* (GCF_022682495.2-RS_2025_02) and genome assemblies of ten additional bat species were retrieved from the National Center for Biotechnology Information (NCBI) database [12] in June 2025. The comparative panel included *Eptesicus fuscus*, *Hipposideros armiger*, *Miniopterus natalensis*, *Myotis brandtii*, *Myotis davidii*, *Myotis lucifugus*, *Phyllostomus discolor*, *Pteropus alecto*, *Pteropus vampyrus*, and *Rousettus aegyptiacus*. To evaluate conservation and divergence, all *D. rotundus* protein sequences were aligned against each reference proteomes using BLASTp (version 2.16) with an E-value threshold of ≤1 × 10^−5^ and a minimum identity of 50%. For broader evolutionary context, the same alignment criteria were applied to three representative model organisms (human, mouse, and rat).

### 2.2. Functional Annotation of Uncharacterized Proteins (UCPs)

In the latest genome assembly of *D. rotundus* (GCF_022682495.2-RS_2025_02), several proteins remain annotated as “uncharacterized proteins” (UCPs), which may conceal important functional factors. To infer putative functions, we first identified homologous proteins as described in Section 2.1 and transferred functional annotations from these homologs to the corresponding UCPs. To ensure reliability, all annotation results were subsequently examined and manually curated, thereby improving the accuracy and biological relevance of the functional assignments. Considering the unique immune adaptations of bats, particular attention was given to UCPs potentially associated with antiviral defense, immune regulation, and host–virus interactions.

### 2.3. Analysis of Interferon Regulatory Factor

Given that *D. rotundus* harbors numerous viruses but typically remains asymptomatic, we hypothesized that lineage-specific adaptations in antiviral proteins may underlie this tolerance. Interferon regulatory factors (IRFs) are key transcriptional regulators that play critical roles in antiviral defense and immune modulation, and recent studies have highlighted their unique evolutionary patterns in bats [13,14]. To investigate this, we retrieved all annotated IRF protein sequences from *D. rotundus*, ten additional bat species, and three representative model organisms (human, mouse, and rat). Pairwise sequence alignments were performed, and identity values were calculated to assess conservation and divergence of IRFs across species.

### 2.4. New miRNA Identification

Because miRNAs form characteristic stem–loop secondary structures, we employed a structure-based prediction pipeline [10,11] to identify novel miRNAs in *D. rotundus*. First, 48,885 mature miRNA sequences were downloaded from miRBase [15] in June 2025, of which 38,371 were annotated as animal miRNAs. All non-coding RNA (ncRNA) sequences of *D. rotundus* were aligned against this reference set using BLASTn (version 2.16). Candidate alignments were filtered using the following criteria: (1) alignment length ≥ 18 nucleotides; (2) mismatch number ≤ 2; and (3) no gaps. For each candidate, 50 nucleotides upstream and downstream of the mapped region were extracted from the ncRNA sequence. These extended sequences were folded with ViennaRNA (version 2.7.0) [16] to calculate the minimum free energy (MFE). Only candidates with MFE ≤ −20 kcal/mol and Ensemble Diversity (ED) ≥ 15 were retained as putative novel miRNAs, as these thresholds ensure both thermodynamic stability and sufficient structural diversity of the predicted stem–loop precursors.

### 2.5. miRNA Target Prediction and Network Construction

We used the miRWalk2.0 database and applied stringent criteria to predict the target genes of the newly identified *D. rotundus* miRNAs. Specifically, only interactions meeting the following thresholds were retained: (1) miRWalk binding score = 1; (2) binding position restricted to the 3′-UTR; and (3) a validated accession number is available. The resulting miRNA–gene interactions were visualized and analyzed as a regulatory network using Cytoscape (version 3.9.1) [17], in which each miRNA was connected to its predicted high-confidence targets. To capture downstream molecular interactions and provide broader functional context, protein–protein association data were retrieved from the STRING database [18] and integrated into the network, thereby enabling a combined view of miRNA-mediated regulation and gene–gene connectivity.

### 2.6. Functional Enrichment of miRNA-Targeted Genes

Functional enrichment of the predicted *D. rotundus* miRNA target genes was performed using the Database for Annotation, Visualization, and Integrated Discovery (DAVID) v6.8 (https://davidbioinformatics.nih.gov/, accessed on 30 June 2025) [19]. Official gene symbols were uploaded as the input list and performed with default parameters. We selected four annotation categories for analysis: GOTERM_BP_DIRECT (Gene Ontology Biological Process), GOTERM_MF_DIRECT (Gene Ontology Molecular Function), GAD_DISEASE (Genetic Association Database disease terms), and KEGG_PATHWAY. Enrichment statistics were computed using DAVID’s built-in modified Fisher’s exact test, with *p*-values < 0.05 considered statistically significant. For each category, the top significant terms were retained for further interpretation, with emphasis on processes and pathways relevant to bat biology, immunity, and antiviral responses.

### 2.7. Genomic Feature Analysis of miRNA-Targeted Genes

The genomic characteristics of miRNA target genes were analyzed using ShinyGO 0.82 (https://bioinformatics.sdstate.edu/go/, accessed on 30 June 2025) [20]. This tool compares user-defined gene sets against the genomic background to assess differences in sequence features such as coding sequence (CDS) length, transcript length, genome span, 3′-UTR length, and GC content. Statistical significance was evaluated using Chi-square and Student’s *t*-tests as implemented in ShinyGO. For each feature, density plots were generated to visualize distribution differences between the miRNA target set and the full genome.

### 2.8. Network Entropy Analysis of Genes

In previous studies, Liu et al. proposed a theory called sample-specific network (SSN) [21] to analyze entropy of individual sample. Inspired by their theory, we introduced a new method called single-factor network to evaluate the importance of individual genes in disease development by calculating the network entropy. Specifically, we removed nodes one at a time, recalculated the network entropy after each removal, and observed changes in the network structure to assess each node’s sensitivity and importance. The network entropy *H* can be calculated by the following Formulas (1) and (2):

(1) $$H=-\overset{N}{\underset{k=1}{\Sigma }}p_{k}\log(p_{k})$$

(2) $$p_{k}=\frac{n_{k}}{N}$$

*H*: network entropy.

*N*: total number of node degree.

$n_{k}$: number of nodes with degree k.

$p_{k}$: the degree probability distribution of the node.

## 3. Results

### 3.1. Protein Homolog Distribution

**Figure 1:**
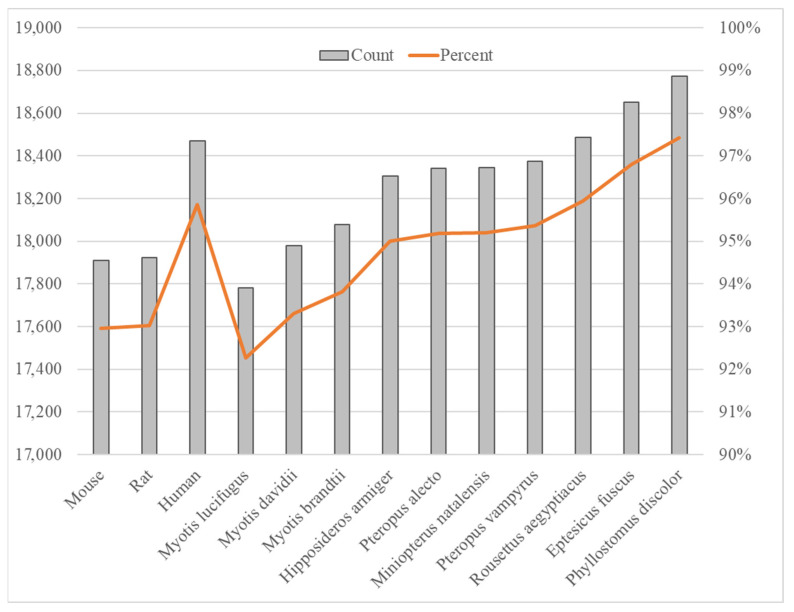
Comparison of *D. rotundus* protein homologs with other species. Ten bats and three model organisms (human, mouse, rat) were compared in this study.

We compared the proteome of *D. rotundus* (annotation version GCF_022682495.2-RS_2025_02) against those of ten other bat species, finding that about 92–98% of *D. rotundus* proteins have identifiable homologs (Figure 1). Specifically, *Phyllostomus discolor* exhibited the highest proteome overlap (97.4%), whereas *Myotis lucifugus* showed the lowest (92.3%). This gradient of homology correlates with known phylogenetic relationships. *P. discolor* is also a New World leaf-nosed bat sharing ecological niches with *D. rotundus*, whereas *M. lucifugus*, a vespertilionid bat widely distributed across Asia, diverged earlier from *D. rotundus*’s lineage [22,23]. Overall, the variation in protein conservation among bat species suggests differing evolutionary pressures, potentially linked to each species’ ecology, such as dietary specialization and roosting behavior. When *D. rotundus* proteins were aligned against those of three model mammals (human, mouse, and rat), only approximately 92–96% of proteins had recognizable homologs, indicating substantial lineage-specific diversification within Chiroptera. Such divergence may underpin bats’ unique immunological adaptations by modifying protein families involved in innate immunity and metabolism.

### 3.2. Function Annotation of UCPs

In the GCF_022682495.2-RS_2025_02 genome annotation release, 540 proteins were initially annotated as “uncharacterized protein”. Through comparative sequence analysis with 10 bats and three model mammals, we successfully annotated 29 previously uncharacterized proteins (UCPs) in *D. rotundus*, converting their generic “uncharacterized” designation into reliable functional assignments (Table 1). All newly annotated proteins exhibited highly significant alignments (E-value ≤ 1 × 10^−8^), with five (XP_045053599.2, XP_071076900.1, XP_024421750.2, XP_053775687.1, XP_053784895.1) matching reference proteins at E = 0. Many of these newly annotated proteins are directly relevant to immune regulation and antiviral defense. Notably, we identified toll-like receptor 4 (TLR4) with high sequence identity (88.57% identity to *Eptesicus fuscus*), a pivotal pattern recognition receptor in sensing bacterial lipopolysaccharide and modulating cross-talk with antiviral interferon pathways [24]. Similarly, the presence of syncytin-1 (94.25% identity to *Phyllostomus discolor*) and endogenous retrovirus group K member 25 (58.58% identity to *Miniopterus natalensis*) underscores the integration of retroviral-derived sequences into the bat genome, which may contribute to placental development and possibly to immune modulation against viral pathogens [25,26]. The identification of both conserved immune effectors TLR4 and retrovirus-derived proteins (syncytin-1, ERVK members) provides new candidates for experimental investigation into the molecular basis of vampire bat viral resilience. These findings will help bridge the gap between genomic annotation and functional immunology, offering a foundation for future studies into bat-specific antiviral strategies.

### 3.3. Similarity of Interferon Regulatory Factor in Mammals

Previous studies [13,14] have shown the importance of interferon regulatory factors (IRFs) in the antiviral mechanisms; thus, we specifically analyzed this protein family. Based on sequence alignment between *Desmodus rotundus* and 13 other mammals, the eight IRFs display a clear split between broadly conserved and highly variable members. The alignment identity of eight IRFs was shown in Table 2. We found their evolution patterns are informative for antiviral specialization in the vampire bat. IRF1, IRF5 and IRF8 are the most conserved: across all species, average identity values are 90.0% (IRF1), 91.7% (IRF5) and 93.6% (IRF8), and several bat lineages show ≥95% identity for these factors. The phyllostomid *Phyllostomus discolor*, phylogenetically closest to *D. rotundus* in our panel, exhibits the highest overall similarity (IRF1 = 99.4%, IRF8 = 96.7%, IRF9 = 94.9%), underscoring strong conservation of core regulatory circuitry within New World leaf-nosed bats. By contrast, more distant non-bat mammals show lower values, particularly for IRF4 and IRF7 (human IRF4 = 42.9%, IRF7 = 64.3%; mouse IRF4 = 39.5%, IRF7 = 58.2%). The antiviral sentinels IRF2, IRF3 and IRF9 are moderately conserved on average, with most bats clustering near the 80–90% range.

In addition, IRF7 is known as the master amplifier of type I interferon responses [27] and shows the greatest cross-species divergence (overall mean = 59.9%) and extreme depressions in several Myotis species (26.4–28.8%). IRF4 is also unusually variable (overall mean = 76.4%), with strikingly low identities in some taxa (*Myotis lucifugus* = 37.7%, human = 42.9%). The exceptionally low cross-species identities for IRF7 and IRF4 pinpoint these factors as priority targets for functional assays in *D. rotundus*. In addition, isolated outliers appear in otherwise conserved genes (*Rousettus aegyptiacus* IRF1 = 44.6%, *Myotis davidii* IRF2 = 45.3%, *Pteropus vampyrus* IRF2 = 49.8%), suggesting lineage-specific divergence in bat evolution. These results indicate that several antiviral modulators of *D. rotundus* exhibit notable sequence divergence and exhibit enhanced sequence plasticity across mammals.

### 3.4. Analysis of New miRNAs 

Using our structure-based pipeline, we identified 19 previously unannotated miRNAs in *D. rotundus*, each met stringent sequence and structural criteria (Table 3). The combination of rigorous filtering parameters, structural verification via ViennaRNA folding, and cross-species conservation strongly supports the authenticity of these predictions. The predicted precursor sequences exhibited minimal free energies (MFE) below −20 kcal/mol, consistent with stable stem-loop secondary structures characteristic of authentic miRNAs, while the calculated ensemble diversities were within expected ranges, supporting reliable secondary structure prediction. In addition, dro-miR-3620-5p and dro-miR-6988-5p displayed strong sequence conservation and highly negative MFE values (−26.6 kcal/mol and −33.1 kcal/mol, respectively), indicating robust stem-loop stability and a high likelihood of functional relevance.

Several identified sequences correspond to highly conserved and functionally important miRNAs known from other vertebrates. For example, dro-let-7-5p, a member of the let-7 family, is widely conserved across mammals and is implicated in antiviral immunity by modulating interferon-stimulated genes [28]. Similarly, dro-miR-296-5p has been reported in previous study as a regulator of inflammatory responses and viral replication [29]. The presence of conserved immune-related miRNAs alongside potentially unique *D. rotundus* sequences offers valuable candidates for functional investigation. These findings provide a strong foundation for exploring miRNA-mediated antiviral regulation in vampire bats.

### 3.5. miRNA Targets and Network Construction

**Figure 2:**
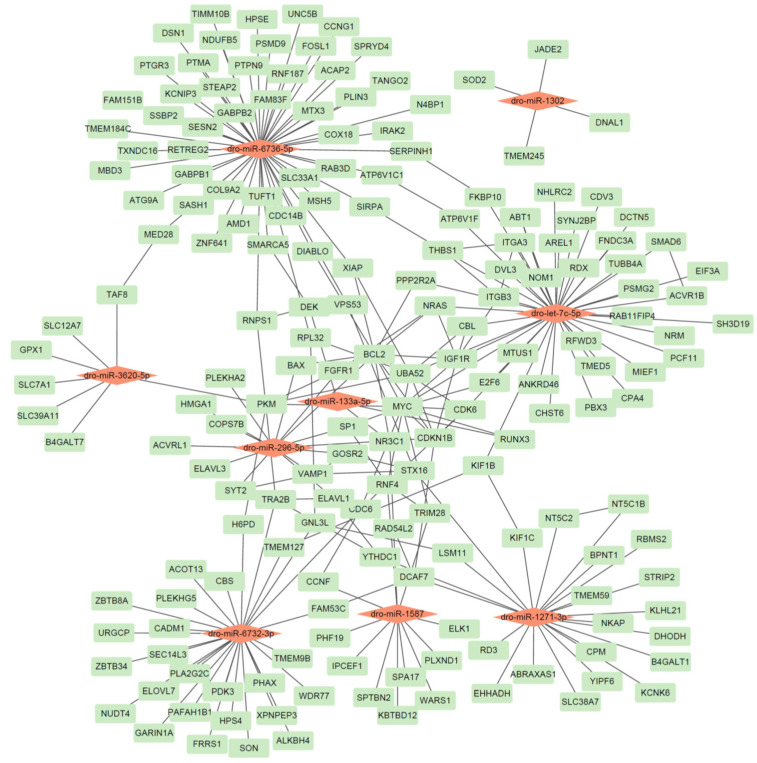
The identified miRNA-gene network in *D. rotundus*. A red node represents a miRNA, while a green node represents a gene.

We identified high-confidence targets for the newly discovered *D. rotundus* miRNAs using miRWalk2.0 and stringent filtering criteria. The top 20 targets were shown in Table 4, and the predicted miRNA–gene interactions network is shown in Figure 2.

Our analysis yielded several key miRNA–gene pairs with strong thermodynamic favorability (binding energies ranging from −17.9 to −37.6 kcal/mol), sufficient seed pairing, and accessible binding sites, underscoring the reliability of our predictions. Several target genes are notably connected to immune or antiviral functions; for example, *SOD2* (targeted by dro-miR-1302; binding energy −17.9 kcal/mol) plays a well-established role in countering oxidative stress during viral infection [30]. *TRIM28* (targeted by dro-miR-1587; −33.6 kcal/mol) and RNF4 (same miRNA; −33.8 kcal/mol) are involved in antiviral innate defense as regulators of transcriptional silencing and ubiquitination pathways [31]. *FGFR1* (targets of dro-miR-296-5p; −32.0 kcal/mol) is implicated in cellular growth and neuronal response pathways that can intersect with antiviral defense and inflammatory regulation [32]. The inclusion of genes such as *SOD2*, *TRIM28*, and *FGFR1* underscores the potential of vampire bat–specific miRNAs to regulate pathways relevant to immune modulation and viral resilience. These findings provide a strong foundation for functional validation of miRNA-mediated antiviral regulation in *D. rotundus*.

### 3.6. Functional Enrichment Analysis of miRNA-Target Genes

Functional enrichment analysis of the miRNA target genes using DAVID revealed several biological processes and pathways that align with known adaptive traits of bats (Table 5). In the biological process (BP) category, significant enrichment was observed for “angiogenesis involved in wound healing” (*p*-value = 8.00 × 10^−5^), and “negative regulation of apoptotic process” (*p*-value = 7.50 × 10^−4^). These processes may contribute to bats’ remarkable tissue repair capacity, which is critical for mitigating damage caused by viral infections. In the molecular function (MF) category, the most enriched term was “protein binding” (85.8%, *p*-value = 1.10 × 10^−7^), alongside more specific functions such as “fibronectin binding” (*p*-value = 2.60 × 10^−3^), and “mRNA binding” (*p*-value = 9.30 × 10^−3^). These functions reflect complex post-transcriptional and extracellular matrix interactions that could support rapid immune modulation and tissue remodeling.

Disease association analysis (GAD) highlighted “aging and longevity” (*p* = 0.021) and the disease class “Infection” (*p*-value = 0.026), consistent with the unusual lifespan and high pathogen tolerance documented in bats. KEGG pathway analysis further identified “Human papillomavirus infection” (*p*-value = 3.00 × 10^−4^), which, while human-specific in name, encompasses antiviral defense and cell cycle control mechanisms that are broadly relevant to bat-virus interactions.

### 3.7. Genomic Feature of miRNA-Targeted Genes

**Figure 3:**
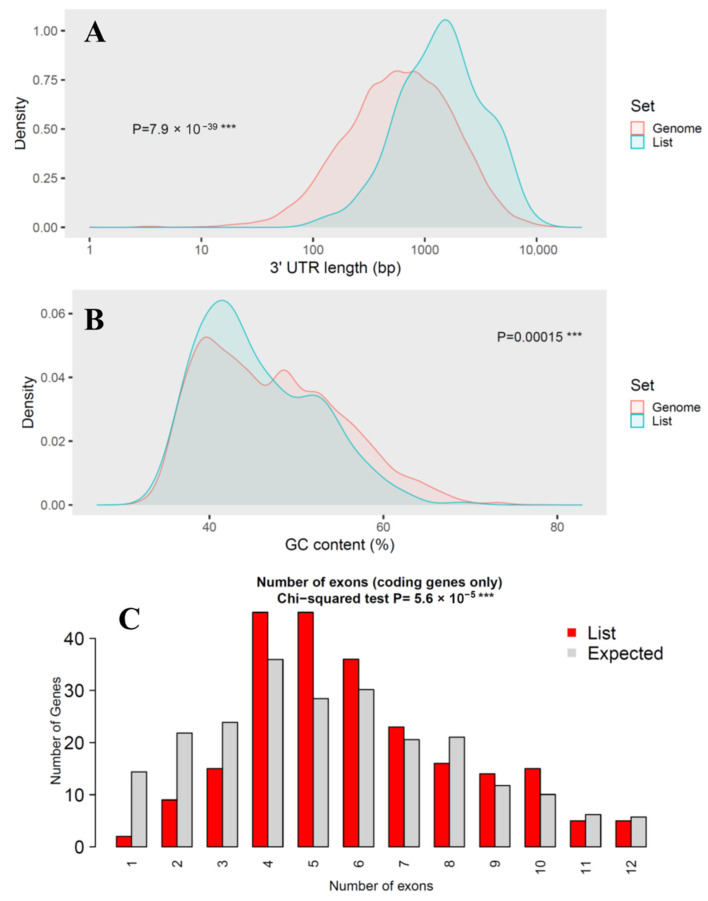
Genomic feature analysis of miRNA target genes. (**A**) Comparison of 3′-UTR length; (**B**) Comparison of GC content; (**C**) Comparison of number of exons. Three consecutive asterisks (***) indicated extremely low *p*-value in statistical test.

To explore the genomic characteristics of the miRNA target genes, we performed gene plot analysis using ShinyGO 0.82 (Figure 3). This approach compares the distribution of various sequence features in our gene set against all annotated genes in the genome, applying the Chi-square test and Student’s *t*-tests for statistical assessment. Our analysis revealed that several genomic features of the target genes differ significantly from the genome-wide background, indicating potential selective constraints and functional specialization.

While coding sequence (CDS) length showed no significant difference (*p*-value = 0.8) between miRNA targets and the genome background, transcript length was markedly longer in the target set (*p*-value = 3 × 10^−12^), suggesting that miRNAs preferentially regulate genes with more extended transcribed regions. This trend was even more pronounced in 3′-UTR length, which was significantly longer in the target genes (*p*-value = 7.9 × 10^−39^), consistent with the canonical miRNA targeting preference for regulatory elements located in extended 3′-UTRs. GC content showed a modest but statistically significant difference (*p* = 0.00015), suggesting potential sequence composition biases in miRNA-regulated genes that could influence mRNA stability or secondary structure. The number of exons also differed significantly between the two groups (*p*-value = 5.6 × 10^−5^), indicating that overall exon size is a major distinguishing factor.

Collectively, these results demonstrate that the *D. rotundus* miRNA target genes are not a random subset of the genome but instead exhibit distinctive sequence characteristics, particularly extended transcript and UTR lengths. Such features are consistent with patterns reported in other mammals and may enhance regulatory plasticity, enabling fine-tuned immune and antiviral responses in vampire bats.

### 3.8. Network Entropy Influenced by Each Gene

Network entropy analysis of the miRNA–gene interaction network identified a subset of genes whose removal produced the largest changes in network entropy, indicating high centrality and functional sensitivity within the regulatory system (Table 6). The top-ranked gene, *MYC* (ΔH = −0.0562), is a master transcription factor regulating cell proliferation, apoptosis, and immune activation, and has been implicated in controlling viral replication and immune cell metabolism [33]. *BCL2* (ΔH = −0.0405) functions as a key anti-apoptotic regulator, potentially contributing to the fine-tuning of cell death during antiviral responses—an adaptation that may help bats limit immunopathology while tolerating persistent viral infections [34]. Other high-impact node *KIF1B*, participates in intracellular transport—a process often targeted by viruses to hijack host machinery [35]. DNA repair and replication control genes such as *RAD54L2*, *CDC6*, and *DEK* also ranked highly, suggesting that genome stability and replication checkpoint regulation are central features in the bat antiviral network [36].

The network entropy results point to a core set of genes integrating cell cycle regulation, apoptosis control, and genome maintenance. These processes are central to balancing viral tolerance and immune defense. These findings align with the known immunological adaptations of bats, highlighting candidate molecular hubs for experimental validation in future antiviral research.

## 4. Discussion

The *Desmodus rotundus* (common vampire bat) exhibits a unique combination of ecological, physiological, and immunological traits that distinguish it from other mammals. As an obligate sanguivore, it is chronically exposed to diverse blood-borne pathogens, including a wide range of viruses, yet it rarely develops overt disease. This adaptability is underpinned by specialized antiviral mechanisms, including constitutively active interferon pathways, positive selection in key immune regulators, and dampened inflammatory responses [6,37]. *D. rotundus* also appears to co-opt endogenous retroviral elements and modulate immune signaling through finely tuned gene regulation, enabling a balance between pathogen control with tissue homeostasis. These adaptations support a tolerance-based antiviral strategy, allowing long-term viral persistence without compromising host fitness. Our multi-layered investigation into *D. rotundus* genomic and regulatory features reveals specific adaptations in bat antiviral immunity, supported by emerging comparative studies.

The functions of 29 uncharacterized proteins were identified in this study. Our annotation of these UCPs, including TLR4, syncytin-1, and ERVK, aligns with the broader notion that bats co-opt viral elements and innate immune receptors to bolster antiviral defenses while dampening inflammatory damage. Indeed, bats are known for dampened inflammasome responses and unique inflammasome sensor loss, which supports tolerance rather than hyperinflammation [38]. The observed high conservation of IRF1, IRF5, and IRF8, contrasted with notable divergence in IRF4 and IRF7, echoes findings by Irving et al. [39], showing elevated basal levels of IRF1/3/7 across bat tissues, which support potent yet controlled interferon-stimulated gene (ISG) responses. Similarly, IRF7’s unique expression patterns and preserved functional domains suggest that bats maintain regulatory flexibility in interferon pathways [14]. Positive selection signatures in IRF family members, including IRF4 and IRF9, have also been observed across bat species, reinforcing their adaptive immunological roles [40].

We also identified 19 novel miRNAs in the genome of *D. rotundus* by applying a set of stringent criteria. This work has highlighted novel miRNAs with homology to immune regulators (dro-let-7-5p, dro-miR-296-5p), converges with global trends where miRNAs mediate host–virus interactions by modulating inflammation, apoptosis, and proliferation. Our target prediction uncovered miRNA regulation of genes, such as *SOD2*, *TRIM28*, and *FGFR1*. These genes were previously implicated in antiviral and oxidative stress pathways. This resonates with studies showing bat immune tolerance involves fine-tuning oxidative stress and DNA repair systems to prevent immunopathology, while maintaining viral resistance. Network entropy analysis spotlighted central hubs (*MYC*, *BCL2* and *KIF1B*), which emerged as key regulators of cell cycle, apoptosis, and signaling. These mirror bat-specific antiviral features, where controlled cell proliferation and survival are balanced to support viral co-existence without disease. Such regulatory resilience echoes proposed mechanisms of bat infection tolerance and longevity [41].

Our integrated results, including sequence evolution, miRNA-mediated regulation, and network centrality, converge with existing literature portraying bats as immunologically adapted species. They retain potent interferon regulation, attenuate inflammatory triggers, leverage endogenous retroelements, and orchestrate precision post-transcriptional regulation. These adaptations allow *D. rotundus* to effectively host viruses while avoiding pathology. Future experimental validation of these miRNAs, IRFs, and hub genes will be crucial to unraveling bat-specific antiviral strategies and their translational potential.

## 5. Conclusions

In summary, our genome-wide analysis of the common vampire bat provides integrative evidence that this species achieves viral tolerance through the combined action of conserved immune factors, lineage-specific protein divergence, and miRNA-mediated fine-tuning of gene expression. The identified immune-related proteins (such as TLR4, syncytin-1, and ERVK members) and newly discovered miRNAs (including dro-miR-296-5p and dro-let-7-5p) represent promising molecular candidates for future functional validation in bat cell systems. Understanding how these regulators maintain immune balance without triggering excessive inflammation may inspire the design of novel antiviral and anti-inflammatory strategies in other mammals. Overall, this study expands current knowledge of bat immunology and provides a foundation for developing bat-informed approaches to enhance viral resistance and immune tolerance in biomedical research.

## Figures and Tables

**Table 1:** Newly annotated UCPs in *D. rotundus*. Currently, these proteins are annotated as “uncharacterized protein” in NCBI database.

DRO Accession	Homolog Species	Our Annotation	Identity(%)	E-Value
XP_045053599.2	mouse	PDZ and pleckstrin homology domains 1	57.21	0
XP_071076900.1	*Rousettus aegyptiacus*	neuronal-specific septin-3 isoform X1	69.84	0
XP_024421750.2	*Phyllostomus discolor*	nascent polypeptide-associated complex subunit alpha	79.30	0
XP_053775687.1	*Rousettus aegyptiacus*	orofacial cleft 1 candidate gene 1 protein	80.74	0
XP_053784895.1	*Phyllostomus discolor*	syncytin-1	94.25	0
XP_045054974.2	*Pteropus vampyrus*	PH domain-containing protein 1	76.39	8.34 × 10^−166^
XP_053784149.1	*Myotis brandtii*	proline-rich receptor-like protein kinase PERK9	80.26	3.66 × 10^−152^
XP_053785850.1	*Miniopterus natalensis*	endogenous retrovirus group K (ERVK) member 25	58.58	2.39 × 10^−142^
XP_045047497.1	*Eptesicus fuscus*	toll-like receptor 4	88.57	4.03 × 10^−134^
XP_071076901.1	*Myotis lucifugus*	kelch domain-containing protein 7B	75.07	7.86 × 10^−132^
XP_053786695.1	*Pteropus alecto*	EF-hand calcium-binding domain-containing protein 3	53.98	9.15 × 10^−132^
XP_024430562.2	*Myotis davidii*	vesicle-associated membrane protein 7	90.34	1.05 × 10^−118^
XP_024407409.2	*Rousettus aegyptiacus*	mitotic spindle assembly checkpoint protein MAD2A	90.45	2.24 × 10^−116^
XP_024414427.2	mouse	thioesterase superfamily member 7	77.39	1.72 × 10^−113^
XP_045052948.3	*Hipposideros armiger*	Polyadenylation specificity factor subunit 5	74.44	6.70 × 10^−110^
XP_045051350.1	*Myotis brandtii*	keratin-associated protein 10	97.84	5.94 × 10^−93^
XP_045058910.2	*Hipposideros armiger*	phospholipase A2 inhibitor	69.49	9.20 × 10^−82^
XP_053771141.1	*Myotis davidii*	protein NYNRIN-like	54.65	2.65 × 10^−76^
XP_053779447.1	*Miniopterus natalensis*	cyclic nucleotide-binding domain-containing protein 2	76.39	7.15 × 10^−69^
XP_053773395.1	*Rousettus aegyptiacus*	doublesex- and mab-3-related transcription factor C2	52.11	4.26 × 10^−53^
XP_045053117.2	*Hipposideros armiger*	sorbin and SH3 domain-containing protein 2	70.73	2.75 × 10^−44^
XP_053766423.1	*Eptesicus fuscus*	ribosomal protein L18	86.42	3.66 × 10^−41^
XP_053779458.1	*Phyllostomus discolor*	translation initiation factor IF-2	62.90	1.94 × 10^−34^
XP_071075791.1	*Myotis brandtii*	sperm acrosome membrane-associated protein 4	77.33	2.14 × 10^−33^
XP_045044110.2	*Eptesicus fuscus*	spermatid nuclear transition protein 3	74.19	7.99 × 10^−29^
XP_071076697.1	*Pteropus vampyrus*	vegetative cell wall protein gp1	82.55	1.90 × 10^−22^
XP_053772343.1	*Hipposideros armiger*	LIM domain only protein	92.86	3.28 × 10^−17^
XP_053769798.1	*Phyllostomus discolor*	collagen alpha-1(I) chain-like	85.00	3.23 × 10^−15^
XP_045047205.2	human	DMRT like family C1	68.18	9.33 × 10^−9^

**Table 2:** The identity value of IRFs in the sequence alignment between *D. rotundus* and other mammals.

Species	IRF1	IRF2	IRF3	IRF4	IRF5	IRF7	IRF8	IRF9
*Eptesicus fuscus*	93.9%	95.1%	83.1%	97.3%	93.7%	73.7%	92.5%	87.5%
*Hipposideros armiger*	95.4%	97.5%	82.4%	96.3%	93.4%	72.5%	93.9%	80.9%
*Miniopterus natalensis*	96.9%	96.0%	81.5%	81.5%	94.6%	75.2%	96.0%	86.2%
*Myotis brandtii*	94.8%	98.7%	81.5%	97.8%	91.9%	28.8%	95.1%	83.8%
*Myotis davidii*	93.9%	45.3%	79.8%	97.8%	91.5%	28.6%	95.3%	83.8%
*Myotis lucifugus*	94.8%	49.5%	81.0%	37.7%	92.3%	26.4%	95.1%	83.0%
*Phyllostomus discolor*	99.4%	96.3%	89.7%	89.8%	95.2%	82.0%	96.7%	94.9%
*Pteropus alecto*	96.7%	97.8%	82.4%	96.9%	92.8%	70.1%	95.1%	86.6%
*Pteropus vampyrus*	96.7%	49.8%	82.2%	78.1%	93.0%	69.8%	94.6%	86.6%
*Rousettus aegyptiacus*	44.6%	96.4%	78.6%	97.1%	92.2%	70.1%	95.1%	85.9%
human	94.8%	96.4%	79.5%	42.9%	87.1%	64.3%	91.3%	78.9%
mouse	83.7%	91.7%	68.6%	39.5%	86.9%	58.2%	89.7%	64.1%
rat	84.6%	86.1%	71.1%	39.9%	87.5%	58.6%	87.1%	63.9%
All species average	90.0%	84.3%	80.1%	76.4%	91.7%	59.9%	93.6%	82.0%

**Table 3:** New identified miRNAs in *D. rotundus*. MFE: minimal free energy; ED: Ensemble Diver-sity. The unit of MFE is kcal/mol.

miRNA	Sequence	Length	Mismatch	MFE	ED
dro-let-7-5p	TGAGGTAGTAGGTTGTATAGTTT	23	0	−23.3	9.55
dro-miR-1271-3p	TGCCTGGCACACAGCAGGCAC	21	0	−28.6	3.82
dro-miR-1291b	GGCCCTGAATCAAGGCCAGCAGT	23	1	−21.3	3.93
dro-miR-1302	TTGGGACATACTTATACTAAA	21	0	−20.9	2.22
dro-miR-133a-5p	AGCTGGTAAAATGGAACCAAAT	22	0	−21.2	4.08
dro-miR-2309	GAGGGTGGTGGAAGGCAGGG	20	1	−27.0	4.81
dro-miR-296-5p	GAGGGCCTCCCTCAACCCTG	20	0	−30.3	14.7
dro-miR-3072	GAAGGCTTCCTGGAGGGGG	19	0	−20.4	4.17
dro-miR-3173	GGCCTGCCTGTGTCCTCCT	19	1	−34.9	2.11
dro-miR-3596	TGAGGTAGTAGGTTGTGTGGTT	22	0	−25.3	4.20
dro-miR-3620-5p	GGCCCAGCCCAGCCCAGCCC	20	0	−26.6	12.38
dro-miR-544-5p	GAATCTGCCTTTTAACAAG	19	0	−22.5	5.59
dro-miR-6732-3p	GGGAGGGGGAGGACAGGGTT	20	1	−22.4	4.58
dro-miR-6736-5p	CTGGGTGAGGGCTTCTGTGG	20	1	−20.1	5.46
dro-miR-6988-5p	GGGCCTCAGCTCACCACCC	19	1	−33.1	10.24
dro-miR-7033-5p	CCAGGGGTCTGAGGGGCA	18	1	−22.4	6.91
dro-miR-8839	TTGGCAGAGCCAGGGTTCAA	20	1	−22.0	3.36
dro-miR-9222	TAGAAGTCCAAACTCAAGGTG	21	1	−21.2	8.38
dro-miR-9993b-3p	TGGAGGCCCCAGCGAGAT	18	0	−20.5	13.67

**Table 4:** miRNA target prediction by miRWalk2.0. The validated ID is retrieved from miRTarBase database. This table only showed top 20 interaction. The full table was provided in Table S1.

miRNA	Targeted Gene	Coding Protein	Binding Energy	Validated ID
dro-let-7c-5p	*TUBB4A*	NM_006087	−25.7	MIRT735640
dro-let-7c-5p	*ABT1*	NM_013375	−23.4	MIRT100093
dro-let-7c-5p	*CHST6*	NM_021615	−24.9	MIRT051817
dro-miR-1271-3p	*CPM*	NM_198320	−36.4	MIRT514569
dro-miR-1271-3p	*LSM11*	NM_173491	−32.3	MIRT522699
dro-miR-1271-3p	*NT5C1B*	NM_033253	−30.7	MIRT451045
dro-miR-1302	*DNAL1*	NM_001201366	−20.3	MIRT688422
dro-miR-1302	*SOD2*	NM_001322814	−17.9	MIRT525780
dro-miR-133a-5p	*RUNX3*	NM_004350	−18.6	MIRT561635
dro-miR-133a-5p	*NR3C1*	NM_001364184	−18.5	MIRT687439
dro-miR-1587	*RNF4*	NM_001185009	−33.8	MIRT121369
dro-miR-1587	*TRIM28*	NM_005762	−33.6	MIRT737922
dro-miR-296-5p	*ELAVL3*	NM_032281	−37.6	MIRT624037
dro-miR-296-5p	*FGFR1*	NM_001354368	−32	MIRT734669
dro-miR-3620-5p	*SLC12A7*	XM_047416640	−37.1	MIRT766116
dro-miR-3620-5p	*TAF8*	XM_017010241	−35.5	MIRT560359
dro-miR-6732-3p	*ALKBH4*	NM_017621	−35.2	MIRT648662
dro-miR-6732-3p	*TMEM9B*	NM_001286094	−31	MIRT446991
dro-miR-6736-5p	*SPRYD4*	NM_207344	−32.2	MIRT607891
dro-miR-6736-5p	*FAM83F*	NM_138435	−30.5	MIRT542970

**Table 5:** Function enrichment of miRNA target genes by DAVID.

Category	Term	Count	Percent	*p*-Value
GOTERM_BP_DIRECT	angiogenesis involved in wound healing	4	2.2%	8.00 × 10^−5^
GOTERM_BP_DIRECT	G1/S transition of mitotic cell cycle	7	3.8%	9.30 × 10^−5^
GOTERM_BP_DIRECT	negative regulation of apoptotic process	14	7.7%	7.50 × 10^−4^
GOTERM_MF_DIRECT	protein binding	157	85.8%	1.10 × 10^−7^
GOTERM_MF_DIRECT	fibronectin binding	4	2.2%	2.60 × 10^−3^
GOTERM_MF_DIRECT	molecular adaptor activity	7	3.8%	9.20 × 10^−3^
GOTERM_MF_DIRECT	mRNA binding	8	4.4%	9.30 × 10^−3^
GAD_DISEASE	aging and longevity	2	1.1%	2.10 × 10^−2^
GAD_DISEASE_CLASS	Infection	33	18.0%	2.60 × 10^−2^
KEGG_PATHWAY	Human papillomavirus infection	13	7.1%	3.00 × 10^−4^

**Table 6:** Network entropy analysis of each gene. New entropy was calculated by removing the corresponding genes in the original network.

Node	Degree	Degree Distribution	Original Entropy	New Entropy	Entropy Change
*MYC*	11	0.0104	1.2716	1.2154	−0.0562
*BCL2*	11	0.0104	1.2716	1.2311	−0.0405
*UBA52*	7	0.0208	1.2716	1.2471	−0.0245
*KIF1B*	3	0.0885	1.2716	1.2486	−0.0230
*THBS1*	4	0.0573	1.2716	1.2496	−0.0220
*STX16*	4	0.0573	1.2716	1.2496	−0.0220
*VAMP1*	4	0.0573	1.2716	1.2496	−0.0220
*RAD54L2*	3	0.0885	1.2716	1.2506	−0.0210
*DEK*	3	0.0885	1.2716	1.2514	−0.0202
*CDC6*	4	0.0573	1.2716	1.2518	−0.0199
*FGFR1*	3	0.0885	1.2716	1.2528	−0.0188
*TRA2B*	4	0.0573	1.2716	1.2541	−0.0176
*CDKN1B*	7	0.0208	1.2716	1.2544	−0.0173
*GNL3L*	3	0.0885	1.2716	1.2546	−0.0171
*CCNF*	2	0.1354	1.2716	1.2551	−0.0166

## Data Availability

The original data presented in this study are openly available in Zenodo repository at https://doi.org/10.5281/zenodo.17351716.

## Supplementary Materials

The following supporting information can be downloaded at https://www.mdpi.com/article/10.3390/ani15213063/s1: Table S1: Full table of miRNA-gene interaction.
